# *Helicobacter pylori*, Betel Quid Chewing, and Endoscopic Pathology in Dyspeptic Patients: A Cross‐Sectional Analytical Study

**DOI:** 10.1002/puh2.70437

**Published:** 2026-11-06

**Authors:** Mostak Uddin Ahmed, Iftekhar Ahmad, Sumona Tanu, Maisha Tanzim Mithila, A. N. M. Ishraqul Haque, Rukaia Tasin Athina, Ratan Chandra Bhowmik, Md. Jahangir Alam, Md. Asaduzzaman

**Affiliations:** ^1^ Department of Gastroenterology Sylhet MAG Osmani Medical College Hospital Sylhet Bangladesh; ^2^ Department of Food Engineering and Tea Technology Shahjalal University of Science and Technology Sylhet Bangladesh; ^3^ Department of Physiology Sylhet MAG Osmani Medical College Sylhet Bangladesh; ^4^ North East Medical College Hospital Sylhet Bangladesh; ^5^ Department of Biochemistry Monno Medical College Manikganj Bangladesh; ^6^ Sylhet MAG Osmani Medical College Sylhet Bangladesh; ^7^ Department of Medicine Sylhet MAG Osmani Medical College Hospital Sylhet Bangladesh

**Keywords:** areca nut, betel quid, dyspepsia, endoscopy, gastritis, *Helicobacter pylori*, interaction analysis, South Asia

## Abstract

**Background:**

*Helicobacter pylori* (*H. pylori*) infection and betel quid (BQ) chewing are both highly prevalent in South Asia and are independently implicated in upper gastrointestinal mucosal injury, yet their joint contribution to endoscopic pathology in dyspeptic patients is poorly characterised. We aimed to describe the exposure profile of dyspeptic patients with and without endoscopic pathology, quantify the individual and joint associations of *H. pylori* and BQ use with pathology and explore trends with BQ exposure.

**Methods:**

In this cross‐sectional analytical study, 211 consecutive dyspeptic patients attending a tertiary gastroenterology department were classified as having endoscopic pathology (Group A, *n* = 141) or a normal endoscopic examination (Group B, *n* = 70). *H. pylori* status, BQ exposure (including a cumulative ‘BQ‐Quid‐Years’ index), and body mass index (BMI) were recorded, and multivariable logistic regression, additive‐interaction analysis and dose–response modelling were performed.

**Results:**

Group A patients were older (median 40 vs. 31 years, *p* = 0.009) and more often male (58.2% vs. 41.4%, *p* = 0.039) than Group B. Pathology prevalence rose stepwise from 52.2% in patients negative for both *H. pylori* and BQ use to 72.5% in patients positive for both (trend *p* = 0.076). The relative excess risk due to interaction (RERI) showed no clear evidence of departure from additivity (−0.16, 95% confidence interval [CI] −1.14 to 0.39), although the wide interval does not exclude synergistic or sub‐additive effects. After multivariable adjustment, male sex (adjusted odds ratio [aOR] 3.01, 95% CI 1.50–6.04, *p* = 0.002) and, more modestly, age (aOR 1.02, 95% CI 1.00–1.05, *p* = 0.039) were independently associated with pathology; *H. pylori* (aOR 1.38, *p* = 0.353) and BQ use (aOR 1.66, *p* = 0.219) showed positive but non‐significant associations.

**Conclusions:**

Male sex was the most robust, independently associated correlate of endoscopic pathology in dyspeptic patients, and age showed a more modest, independent association.

## Graphical abstract

In dyspeptic patients, Helicobacter pylori infection and betel quid chewing showed graded but non‐significant associations with endoscopic pathology, with their joint effect suggesting additivity rather than synergy. Longer betel quid chewing and tobacco use showed suggestive dose–response associations.

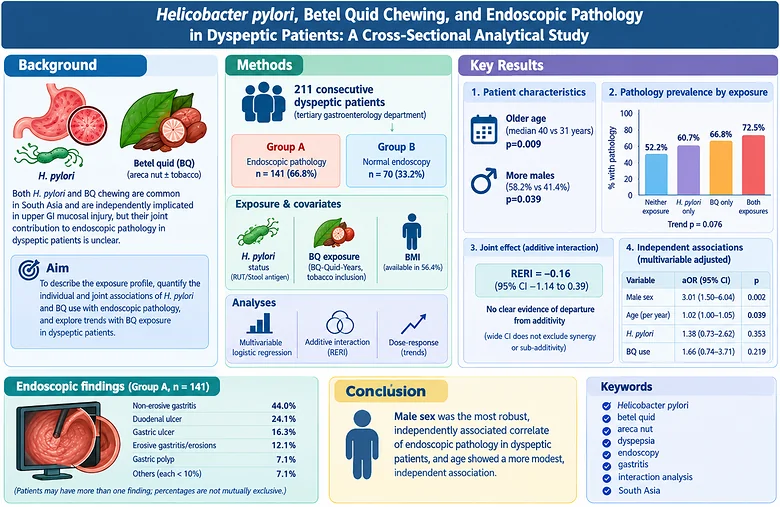

## 1 Introduction

Dyspepsia, a symptom complex of epigastric pain, postprandial fullness, early satiety and epigastric burning, is one of the most frequent reasons for gastroenterology referral worldwide and is formally defined by the Rome IV criteria for disorders of gut–brain interaction [1, 2]. Population‐based surveys applying the Rome IV symptom criteria estimate that roughly 1 in 10 adults in North American and European cohorts meet the definition of functional dyspepsia [3], whereas organic and structural causes of dyspepsia, including chronic gastritis, peptic ulcer disease and, less commonly, gastric malignancy, remain considerably more prevalent among patients undergoing diagnostic upper gastrointestinal endoscopy in South Asian settings [4]. Distinguishing patients with clinically important endoscopic pathology from those with functional symptoms is therefore a central task in dyspepsia management, and identifying the exposures that drive this distinction has direct implications for endoscopy referral thresholds and preventive counselling.

*Helicobacter pylori* remains the single most important identifiable cause of chronic gastritis, peptic ulcer disease and gastric cancer. Despite a well‐documented secular decline in prevalence over the past four decades, the infection is still estimated to affect over 40% of the world's adult population, with the highest burden persisting in low‐ and middle‐income countries [5]. Among *H. pylori*‐infected individuals, the pooled global prevalence of gastric cancer has been estimated at 12%–18%, with marked regional variation and the highest rates reported in East Asia [6]. Bangladesh and the wider South Asian subcontinent continue to report some of the highest *H. pylori* prevalence figures internationally, with pooled estimates for asymptomatic populations reaching 86% in Bangladeshi samples [7]. Hospital‐based endoscopy series from Dhaka and Mymensingh report infection rates of roughly one‐third to one‐half of dyspeptic patients tested by rapid urease testing, stool antigen assay or polymerase chain reaction (Tanni et al. 2021; [8, 9]), and even chronically malnourished pre‐school children in Dhaka slums show endoscopically confirmed *H. pylori*‐associated duodenitis [10]. A Bangladeshi case–control study has directly linked *H. pylori* infection to gastric cancer risk in this population [11], and gastritis, the most frequent endoscopic finding among dyspeptic South Asian patients—together with duodenal ulcer—is consistently associated with *H. pylori* positivity across this literature [8, 12]. Whether *H. pylori* acts as an independent risk factor for pathology also depends on host characteristics: Pooled international data indicate a modest but consistent male excess in infection prevalence [13], whereas the relationship between *H. pylori* and body mass index (BMI) is inconsistent across populations, with some studies reporting an inverse association and others no association at all [14].

A second, less well‐characterised exposure of regional importance is betel quid (BQ) chewing, a preparation of areca nut, betel leaf and slaked lime, frequently combined with tobacco products such as zorda or sadapata, consumed by an estimated 600 million people worldwide, predominantly across South and Southeast Asia [15]. BQ, whether or not it contains tobacco, together with areca nut itself, is classified by the International Agency for Research on Cancer as a Group 1 human carcinogen on the basis of consistent evidence for oral, pharyngeal and oesophageal cancer [16]. Case–control and cohort data from Taiwan and the Indian subcontinent report substantially elevated risks of oral premalignant and malignant lesions among habitual chewers, rising further with concurrent tobacco and alcohol use [15, 17]. National tobacco‐use surveys confirm that BQ, frequently combined with smokeless tobacco, remains the most common form of tobacco use among Bangladeshi adults [18]. Chronic BQ exposure is not confined to the oral cavity: Quid juice and dissolved alkaloids, such as arecoline, are swallowed and bathe the oesophageal and gastric mucosa throughout the day, and hospital‐based series of habitual chewers report an excess of reflux oesophagitis, gastric ulcer and duodenitis compared with non‐chewers, with risk increasing further when chewing is combined with smoking and alcohol use [19, 20]. Despite this, the independent contribution of BQ exposure to gross endoscopic pathology in dyspeptic patients, as distinct from its well‐established oral and oesophageal carcinogenicity, has rarely been quantified using a cumulative dose metric analogous to pack‐years for smoking.

Because *H. pylori* infection and BQ use frequently co‐occur in South Asian populations, and because both exposures plausibly injure the gastroduodenal mucosa through overlapping inflammatory and chemical mechanisms, a clinically important question is whether the two exposures act merely additively or instead interact synergistically to raise the risk of endoscopically detectable pathology. Formal statistical tests of additive interaction, such as the relative excess risk due to interaction (RERI), provide a principled framework for distinguishing these possibilities and are increasingly recommended over simple stratified comparisons when evaluating the joint effect of two exposures [15, 21, 22]. Existing evidence addressing this specific combination is sparse: Published work has generally examined either *H. pylori* and dyspepsia or BQ and oral/oesophageal cancer in isolation, and a formal interaction analysis of the joint exposure has, to our knowledge, not previously been reported for endoscopic gastroduodenal pathology in a South Asian dyspepsia cohort.

We therefore conducted a cross‐sectional analytical study of patients presenting with dyspepsia to a tertiary gastroenterology department, with the aims of (i) describing and comparing the demographic, anthropometric and exposure profile of patients with and without endoscopic pathology; (ii) quantifying the individual and joint associations of *H. pylori* infection and BQ use with pathology prevalence, including formal testing for additive interaction; (iii) identifying independent predictors of pathology in multivariable models adjusted for age, sex, BMI, *H. pylori* status and BQ use and (iv) exploring trends with BQ exposure.

## 2 Methods

### 2.1 Study Design and Setting

This was a cross‐sectional analytical study conducted among patients presenting consecutively with dyspepsia to the gastroenterology outpatient department of a tertiary‐care hospital. All participants underwent diagnostic upper gastrointestinal endoscopy, and gross endoscopic findings were used to classify patients into Group A (dyspepsia with endoscopic pathology) or Group B (dyspepsia without endoscopic pathology, i.e., a macroscopically normal upper gastrointestinal tract). Endoscopic pathology (Group A) was defined as any macroscopically visible structural abnormality of the oesophagus, stomach or duodenum at upper gastrointestinal endoscopy, including non‐erosive gastritis, erosive gastritis/erosions, gastric ulcer, duodenal ulcer, duodenitis, gastric polyps, suspected malignancy, deformed duodenal bulb/outlet obstruction, Barrett's oesophagus, oesophagitis, hiatus hernia and xanthoma. Group B comprised patients whose examination was recorded as normal. Classification followed the Rome IV conceptual framework for distinguishing organic from functional gastroduodenal disease, under which the presence of a structural lesion on endoscopy precludes a diagnosis of functional dyspepsia [1].

### 2.2 Participants

A total of 211 consecutive dyspeptic patients were recruited. Of these, 141 (66.8%) were classified as Group A (pathology) and 70 (33.2%) as Group B (normal endoscopy).

### 2.3 Exposure and Covariate Definitions

*H. pylori* status was defined as positive if either the rapid urease test (RUT) or the stool antigen test at baseline was positive, and negative if both available tests were negative. All 211 participants underwent both tests. RUT was positive in 109 (51.7%) and negative in 102 (48.3%); stool antigen was positive in 89 (42.2%) and negative in 122 (57.8%). The two tests were concordant in 171 patients (81.0%) and discordant in 40 (19.0%; RUT‐positive/stool‐negative in 30, RUT‐negative/stool‐positive in 10; Cohen's *κ* = 0.62). Applying the pre‐specified rule yielded 119 *H. pylori*‐positive patients (56.4%) and 92 negative (43.6%), with no indeterminate or missing cases (Table S1). The frequency of biopsy procedures stratified by endoscopic findings and *H. pylori* status is detailed in Table S2.

BQ user status was defined as any lifetime history of BQ use (yes/no). A composite cumulative‐dose index, the ‘BQ‐Quid‐Years’—analogous to pack‐years for cigarette smoking—was derived as duration of use in years multiplied by the average number of quids consumed per day. Inclusion of tobacco within the quid (e.g., zorda and sadapata) was coded from the patient‐reported form of consumption.

BMI was obtained from recorded values where available or calculated from height and weight measurements in the dataset (kg/m^2^). BMI data were available for 119 of 211 participants (56.4%), whereas 92 (43.6%) had missing BMI because height and/or weight measurements were unavailable in the source records. Given the substantial proportion of missing anthropometric data, BMI was excluded from the primary multivariable analysis and evaluated in a reduced‐sample sensitivity analysis. Participants with and without BMI data did not differ significantly in pathology‐group status (67.2% vs. 66.3% Group A, *p* = 1.00), *H. pylori* status (*p* = 0.196), age (*p* = 0.072), sex (*p* = 0.084) or residence (*p* = 0.403) but were significantly less likely to be current betel‐quid users if BMI was missing (66.7% vs. 81.5%, *p* = 0.031); the BMI‐adjusted sensitivity analysis should, therefore, be interpreted with some caution. Smoking status, available for 150/211 participants (71.1%), and alcohol consumption, which was not recorded in this dataset, were considered as additional potential confounders (Tables S3 and S4).

### 2.4 Statistical Analysis

Statistical analyses were performed using Python 3 (pandas, NumPy, SciPy and statsmodels). Continuous variables were assessed for normality using the Shapiro–Wilk test and compared using the independent‐samples t‐test or Mann–Whitney U test, as appropriate. Categorical variables were compared using the chi‐square test or Fisher's exact test when expected cell counts were small. Trends across ordered exposure categories were evaluated using the Cochran–Armitage trend test. Concordance between the two *H. pylori* diagnostic tests (RUT and stool antigen test) was assessed using Cohen's *κ*. Multivariable logistic regression was used to estimate adjusted odds ratios (aORs) and 95% confidence intervals (CIs) for endoscopic pathology after adjustment for age, sex, H. pylori status and BQ use, with BMI and smoking status each added in separate sensitivity models. Because BMI data were unavailable for 92 of 211 participants (43.6%), the primary multivariable model excluded BMI to maximise sample size, with a sensitivity analysis, including BMI in participants with available anthropometric data. The joint association of H. pylori infection and BQ use with endoscopic pathology was evaluated across four exposure groups (neither exposure, H. pylori only, BQ only and both exposures) using the doubly unexposed group as the reference. Overall group differences were assessed using the chi‐square test, pairwise comparisons using Fisher's exact test, and exposure–outcome trend analysis of BQ among BQ users using the Cochran–Armitage trend test. Additive interaction between H. pylori infection and BQ use was assessed by calculating the RERI with a 95% CI obtained by non‐parametric bootstrap (10,000 resamples) given the modest size of some exposure subgroups. No formal a priori sample‐size calculation was performed; the analytic sample reflects consecutive recruitment of dyspeptic patients over the study period. A two‐sided *p* value <0.05 was considered statistically significant.

## 3 Results

### 3.1 Baseline Characteristics (Tables 1 and 2)

Patients with endoscopic pathology (Group A, *n* = 141) (Tables S4 and S5) were older than those without pathology (Group B, *n* = 70) (median 40.0 vs. 31.0 years, *p* = 0.009) and were more frequently male (58.2% vs. 41.4%, *p* = 0.039). BMI, *H. pylori* positivity, BQ use, tobacco inclusion in the quid, duration of BQ use, cumulative BQ‐Quid‐Years and rural residence did not differ significantly between groups (all *p* > 0.05) (Table 1).

**TABLE 1:** Baseline characteristics of dyspeptic patients with (Group A) and without (Group B) endoscopic pathology.

Variable	Group A (*n* = 141)	Group B (*n* = 70)	*p* value
Age, years—median (IQR)	40.0 (30.8–56.5)	31.0 (25.0–51.8)	0.009^*^
BMI, kg/m^2^—mean ± SD	20.7 ± 4.2	21.4 ± 3.7	0.337
Duration of BQ use, years—mean ± SD	23.7 ± 15.8	18.5 ± 15.5	0.109
BQ‐Quid‐Years index—mean ± SD	127.4 ± 171.8	86.8 ± 96.9	0.193
Sex—male/female, *n*	82/57	29/39	0.039^*^
*Helicobacter pylori* positive, *n* (%)	83 (58.9)	36 (51.4)	0.380
Betel quid user, *n* (%)	102 (79.1)	43 (69.4)	0.197
Tobacco included in quid (among BQ users), *n* (%)	58 (55.2)	21 (46.7)	0.432
Residence—Rural, *n* (%)	103 (81.1)	51 (82.3)	1.000

*Note:* Continuous variables compared with the Mann–Whitney *U* test (age, BQ‐duration and BQ‐Quid‐Years) or independent *t*‐test (BMI); categorical variables compared with the chi‐square test. Denominators vary by variable owing to missing data (see Section 2).

Abbreviations: BMI, body mass index; BQ, betel quid; IQR, interquartile range; SD, standard deviation.

* *p* < 0.05.

Among the 141 patients classified as having endoscopic pathology (Group A), non‐erosive gastritis was the most frequently observed finding (62/141, 44.0%), followed by duodenal ulcer (34/141, 24.1%), gastric ulcer (23/141, 16.3%) and erosion/erosive gastritis (17/141, 12.1%). Other findings included gastric polyps (7.1%), malignancy or suspicious lesions (4.3%), deformed bulb/outlet obstruction (3.5%) and duodenitis (2.1%). Less frequent findings included Barrett's oesophagus (1.4%), oesophagitis (0.7%), hiatus hernia (0.7%) and xanthoma (0.7%). Three patients (2.1%) had other or unclassified endoscopic findings (Table 2).

**TABLE 2:** Frequency of endoscopic finding among Group A.

Endoscopic finding (Group A, *n* = 141)	*n*	%
Gastritis (non‐erosive)	62	44.0
Duodenal ulcer	34	24.1
Gastric ulcer	23	16.3
Erosion/Erosive gastritis	17	12.1
Gastric polyp	10	7.1
Malignancy/Suspicious lesion	6	4.3
Deformed bulb/outlet obstruction	5	3.5
Duodenitis	3	2.1
Other/Unclassified text	3	2.1
Barrett's oesophagus	2	1.4
Oesophagitis	1	0.7
Hiatus hernia	1	0.7
Xanthoma	1	0.7

### 3.2 Combined Effect of *H. pylori* and BQ Exposure (Table 3)

The prevalence of endoscopic pathology increased progressively across combined exposure groups, from 52.2% among participants negative for both *H. pylori* and BQ use to 72.5% among those positive for both exposures. Although the odds of pathology were highest in the doubly exposed group (OR 2.42), neither the overall group comparison (*p* = 0.330) nor the Cochran–Armitage trend test (*p* = 0.076) reached statistical significance. The RERI was −0.16 (95% CI −1.14 to 0.39), indicating no clear evidence of departure from additivity; however, the wide interval, which crosses zero, is compatible with both sub‐additive and synergistic interaction, so the analysis is inconclusive (Table 3).

**TABLE 3:** Combined effect of *Helicobacter pylori* and betel quid (BQ) use on endoscopic pathology prevalence.

Group	*n*	Pathology (*n*)	Prevalence (%)	OR vs. reference	*p* value
1. *H. pylori*(−)/BQ non‐user (Reference)	23	12	52.2	1.00 (ref)	—
2. *H. pylori*(+)/BQ non‐user	23	15	65.2	1.72	0.550
3. *H. pylori*(−)/BQ user	65	44	67.7	1.92	0.213
4. *H. pylori*(+)/BQ user	80	58	72.5	2.42	0.079

*Note:* Overall 4 × 2 chi‐square = 3.43, df = 3, *p* = 0.330. Cochran–Armitage trend test across ordered groups (1 → 4): *Z* = 1.77, *p* = 0.076. Relative excess risk due to interaction (RERI) = −0.16 (95% CI −1.14 to 0.39, non‐parametric bootstrap) (component risk ratios: *H. pylori*‐only 1.25, BQ‐only 1.30, combined 1.39), all 211 participants had a determinate *H. pylori* status (Section 2.3); 20 patients with missing betel‐quid exposure status were excluded, leaving an analytic *n* = 191.

Abbreviations: BQ, betel quid; OR, odds ratio (Fisher's exact test vs. Group 1).

### 3.3 Multivariable Logistic Regression (Table 4A, 4B, 4C)

In the primary multivariable model (Table 4A), male sex was independently associated with endoscopic pathology (aOR 3.01, 95% CI 1.50–6.04, *p* = 0.002). Age was also independently associated with pathology (aOR 1.02 per year, 95% CI 1.00–1.05, *p* = 0.039), whereas *H. pylori* positivity (aOR 1.38, *p* = 0.353) and BQ use (aOR 1.66, *p* = 0.219) were not independently associated with pathology. In the BMI‐adjusted sensitivity analysis (Table 4B), male sex remained the only significant predictor (aOR 2.58, 95% CI 1.03–6.45, *p* = 0.043), whereas BMI was not associated with pathology. In a further sensitivity analysis (Table 4C) restricted to the subgroup with smoking data (*n* = 124, 89 events), male sex remained the strongest correlate (aOR 4.09, 95% CI 1.66–10.08, *p* = 0.002), and smoking itself was not significantly associated with pathology (aOR 0.80, 95% CI 0.34–1.91, *p* = 0.620); age attenuated to non‐significance in this smaller subgroup (aOR 1.01, *p* = 0.335), most likely reflecting reduced power.

**TABLE 4A:** Multivariable logistic regression—primary model (age, sex, *Helicobacter pylori* and BQ use).

Predictor	Adjusted OR	95% CI	*p* value
Age (per year)	1.02	1.00–1.05	0.039
Sex (male vs. female)	3.01	1.50–6.04	0.002*
*H. pylori* positive (vs. negative)	1.38	0.70–2.73	0.353
BQ user (vs. non‐user)	1.66	0.74–3.74	0.219

*Note:* Complete‐case *n* = 175, 119 events. Likelihood‐ratio *χ* ^2^ = 17.61, df = 4, *p* = 0.0015; McFadden pseudo‐*R* ^2^ = 0.080.

Abbreviations: BQ, betel quid; CI, confidence interval; OR, odds ratio.

* *p* < 0.05.

**TABLE 4B:** Multivariable logistic regression—sensitivity model, adding BMI.

Predictor	Adjusted OR	95% CI	*p* value
Age (per year)	1.02	0.99–1.05	0.222
Sex (male vs. female)	2.58	1.03–6.45	0.043*
BMI (per kg/m^2^)	0.98	0.88–1.10	0.732
*Helicobacter pylori* positive (vs. negative)	0.95	0.40–2.29	0.911
BQ user (vs. non‐user)	1.87	0.60–5.78	0.279

*Note:* Complete‐case *n* = 106, 72 events. Likelihood‐ratio *χ* ^2^ = 8.75, df = 5, *p* = 0.120; McFadden pseudo‐*R* ^2^ = 0.066.

Abbreviations: BMI, body mass index; BQ, betel quid; CI, confidence interval; OR, odds ratio.

* *p* < 0.05.

**TABLE 4C:** Multivariable logistic regression—sensitivity model, adding smoking.

Predictor	Adjusted OR	95% CI	*p* value
Age (per year)	1.01	0.99–1.04	0.335
Sex (male vs. female)	4.09	1.66–10.08	0.002*
*Helicobacter pylori* positive (vs. negative)	1.86	0.80–4.31	0.150
BQ user (vs. non‐user)	1.65	0.57–4.82	0.358
Smoking (yes vs. no)	0.80	0.34–1.91	0.620

*Note:* Complete‐case *n* = 124, 89 events. Likelihood‐ratio *χ* ^2^ = 13.41, df = 5, *p* = 0.020; McFadden pseudo‐*R* ^2^ = 0.091. Alcohol‐use data were not available in this dataset.

Abbreviations: BQ, betel quid; CI, confidence interval; OR, odds ratio.

* *p* < 0.05.

### 3.4 Exposure–Outcome Trend Analysis of BQ Among BQ Users (Table 5, Figure 1)

Among BQ users, continuous measures of exposure (BQ‐Quid‐Years and chewing‐session duration) were not significantly associated with ulcer or erosion on endoscopy, showing no evidence of a dose–response relationship. However, ulcer/erosion prevalence increased across chewing‐session‐duration tertiles (20.5%, 26.5% and 38.9%), with a suggestive borderline trend (*p* = 0.054). Tobacco inclusion in the quid was associated with a higher prevalence of ulcer/erosion (36.2% vs. 21.4%, *p* = 0.082), although this did not reach statistical significance.

**TABLE 5:** Exposure–outcome trend analysis of betel quid among BQ users (*n* = 145).

Exposure metric (per‐unit OR)	*n* (events)	OR	95% CI	*p* value
BQ‐Quid‐Years index (per unit)	94 (23)	1.001	0.998–1.004	0.468
Duration per chewing session, min (per minute)	114 (32)	1.005	0.977–1.034	0.740
Combined model—BQ‐Quid‐Years	93 (23)	1.001	0.998–1.004	0.567
Combined model—session duration (min)	93 (23)	1.006	0.973–1.039	0.734

*Note:* Outcome = the presence of ulcer or mucosal erosion on gross endoscopy (a narrower definition than overall Group A pathology). Ulcer/erosion prevalence by chewing‐session‐duration tertile (Cochran–Armitage trend *Z* = 1.93, *p* = 0.054): low (≤5 min) 20.5% (9/44); medium (5–10 min) 26.5% (9/34) and high (>10 min) 38.9% (14/36). Pathology rate by tobacco inclusion: for ulcer/erosion, 36.2% (25/69) with tobacco vs. 21.4% (15/70) without (*χ* ^2^ = 3.03, *p* = 0.082); for any endoscopic pathology, 75.7% (53/70) vs. 66.2% (47/71) (*χ* ^2^ = 1.12, *p* = 0.290).

Abbreviations: BQ, betel quid; CI, confidence interval; OR, odds ratio.

**FIGURE 1:**
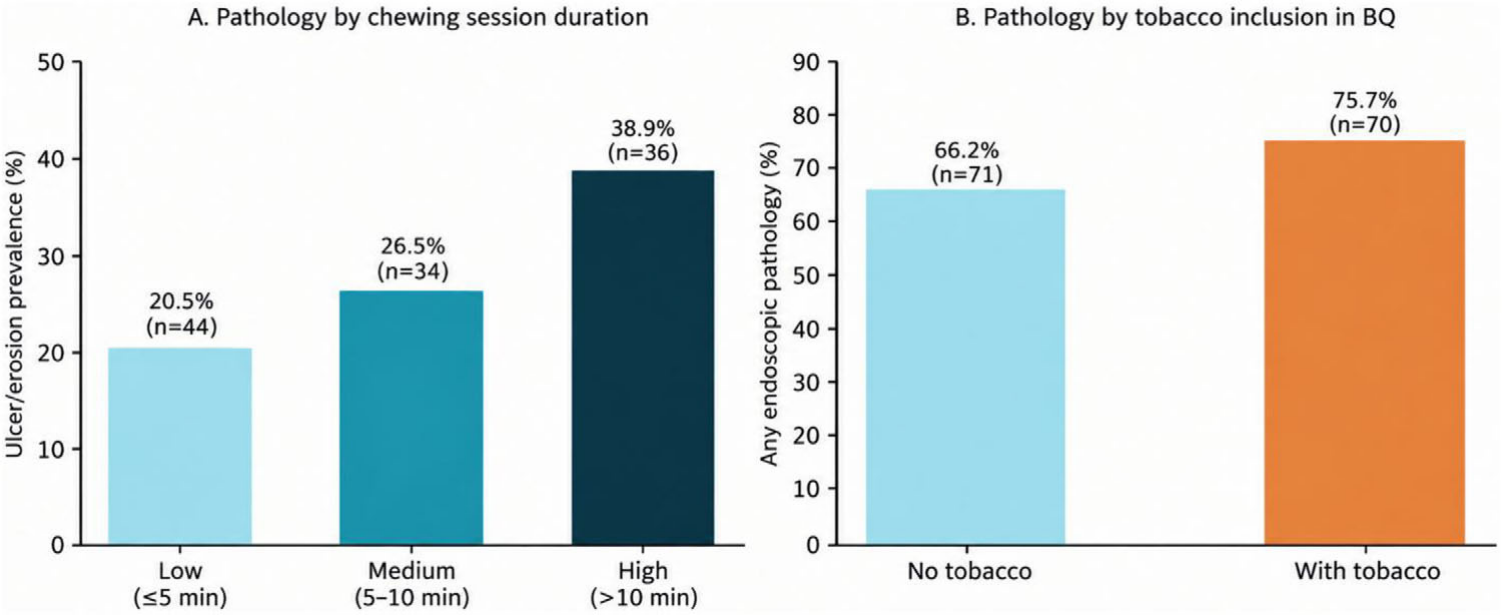
(A) Ulcer/erosion prevalence by betel‐quid chewing‐session‐duration tertile among BQ users. (B) Prevalence of any endoscopic pathology by tobacco inclusion in the betel quid, among BQ users. BQ, betel quid.

## 4 Discussion

In this cross‐sectional analytical study of 211 dyspeptic patients undergoing diagnostic upper gastrointestinal endoscopy, endoscopic pathology was present in two‐thirds of patients and was independently associated with older age and, more strongly, with male sex. *H. pylori* infection and BQ use each showed a positive, biologically plausible, but statistically non‐significant association with pathology after multivariable adjustment, and their combined effect across four exposure strata rose in a graded fashion that approached but did not reach significance for trend. Among BQ users, ulcer/erosion prevalence showed a suggestive, non‐significant upward trend across chewing‐session‐duration tertiles, whereas continuous exposure measures and tobacco inclusion showed no statistically significant association. The male predominance of endoscopic pathology observed here (adjusted odds approximately three‐fold higher than in women) is consistent with the broader epidemiology of *H. pylori*‐related gastroduodenal disease, in which men experience a modestly higher prevalence of infection internationally [13] as well as a disproportionate share of the downstream complications of peptic ulcer disease and gastric cancer. Because male sex remained significant in both the primary and BMI‐adjusted models, whereas *H. pylori* positivity itself was not independently associated with pathology, our findings suggest that sex‐linked factors in this cohort, potentially including unmeasured behavioural exposures such as smoking or alcohol use that are more prevalent among male patients in South Asian settings, may be operating partly independently of *H. pylori* status, an interpretation that is consistent with reports that concurrent tobacco, alcohol and BQ use compound the risk of upper digestive tract disease well beyond that of any single exposure [19].

The absence of a statistically significant independent association between *H. pylori* positivity and endoscopic pathology after adjustment, despite a numerically higher prevalence in Group A at the univariate level, should be interpreted cautiously rather than as evidence against a causal role for the organism. *H. pylori* is a well‐established cause of chronic gastritis and peptic ulcer disease in the wider literature, including in Bangladeshi cohorts where the organism has been directly linked to gastric cancer risk [11] and where gastritis and duodenal ulcer are consistently over‐represented among infected dyspeptic patients [8, 9]. The comparatively modest and non‐significant aOR observed in our sample (1.38) may instead reflect the composition of the ‘no pathology’ comparison group, which in a cross‐sectional endoscopy‐referred sample is itself likely to be enriched for milder or resolving mucosal inflammation not always distinguishable from truly normal mucosa on gross inspection alone, together with the fact that *H. pylori*‐positive patients with normal endoscopy were essentially never biopsied in this cohort (0 of 36 *H. pylori*‐positive Group B patients underwent biopsy, vs. 72.3% of Group A patients overall), so the true burden of microscopic gastritis in this subgroup, and hence potential outcome misclassification, could not be assessed.

BQ use showed a numerically consistent positive association with pathology across every analysis in this study, univariate comparison, the four‐way combined‐exposure table, and both multivariable models, yet did not reach significance in any single test, most plausibly reflecting limited power for a moderate effect size (aOR approximately 1.9) rather than a true absence of association. This pattern is broadly consistent with the wider literature on BQ and upper gastrointestinal disease: Habitual BQ chewers have been reported to carry a two‐fold increased risk of gastric ulcer and duodenitis compared with non‐chewers, particularly when chewing is combined with tobacco and alcohol use [19], and biological plausibility is provided by the direct gastric exposure to swallowed arecoline and related alkaloids, which are established Group 1 carcinogens and mucosal irritants of the upper aerodigestive and gastrointestinal tract [16, 17]. The upward trend across duration tertiles is hypothesis‐generating only; continuous models showed no association, and this pattern may reflect chance or a non‐linear effect, even though the corresponding continuous per‐unit models were underpowered to detect it, a discrepancy that likely reflects both the smaller analytic sample available once restricted to BQ users with complete dosing data and a possible threshold, rather than strictly linear, biological effect of prolonged mucosal contact time.

A central and, to our knowledge, novel contribution of this analysis is the formal test of additive interaction between *H. pylori* infection and BQ use. Although pathology prevalence increased across exposure groups to 72.5% among those positive for both exposures, the RERI (−0.16, 95% CI −1.14 to 0.39) showed no clear evidence of departure from additivity. This finding contrasts with the well‐documented multiplicative interaction between tobacco, alcohol and BQ in the pathogenesis of oral and oesophageal cancer, where combined habitual use has been reported to increase risk more than 17‐fold relative to abstainers [17, 19], a pattern that may reflect the shared, spatially co‐localised mucosal target (the oral cavity and oesophagus) and the specifically genotoxic, nitrosamine‐mediated mechanism of areca‐nut carcinogenesis in that setting [16]. In the stomach, by contrast, *H. pylori*‐induced injury operates through a distinct, bacterially mediated inflammatory pathway that may act largely independently of the chemical irritant effects of swallowed BQ constituents, which could be consistent with largely independent mechanisms, although our data cannot confirm this. Clinically, an additive rather than multiplicative interaction means that the excess risk from dual exposure is proportional to the combined effects of the two exposures, rather than being disproportionately increased by their combination. This finding is directly relevant to risk communication and can help prioritise modifiable exposures for intervention in patients with dual exposure.

These findings should be interpreted in light of several limitations. First, BMI was missing in 43.6% of patients, substantially reducing the sample and hence the statistical power available for the BMI‐adjusted sensitivity model; participants with and without BMI data were otherwise broadly similar, though those with missing BMI were somewhat less likely to be current betel‐quid users (66.7% vs. 81.5%, *p* = 0.031); although a data‐quality check comparing directly reported and calculated BMI values in the subset with both showed close agreement, supporting the validity of our imputation‐by‐calculation approach, residual uncertainty around the BMI‐adjusted estimates remains [22]. Second, several BQ dosing variables—duration, frequency and quantity, were captured as free text and required parsing, with 40%–55% missingness for some derived indices, which will have reduced power for the dose–response analyses in particular. Third, histopathological confirmation was available for only 104 of 211 patients (49.3%), concentrated overwhelmingly in Group A (72.3%) rather than Group B (2.9%); notably, none of the 36 *H. pylori*‐positive Group B patients were biopsied, so some misclassification of ‘pathology’ versus ‘normal’ mucosa at the histological level cannot be excluded, particularly among *H. pylori*‐positive patients with grossly normal endoscopy. Fourth, the cross‐sectional design precludes causal inference and is subject to the general limitations of endoscopy‐referred samples, including possible referral and Berkson‐type selection bias. Fifth, although the overall sample size was reasonable for the baseline and combined‐exposure comparisons (Tables 1 and 2), it limited statistical power for the more granular, stratified dose–response modelling of BQ exposure (Table 4), and no formal a priori sample‐size calculation was performed. Sixth, data on alcohol consumption were not collected; smoking status, available for 150/211 patients, was examined in a sensitivity analysis and did not itself predict pathology, but residual confounding by unmeasured alcohol use cannot be excluded. Finally, as with any single‐centre hospital‐based study, generalisability to community‐based or other regional populations should be considered cautiously, particularly given the substantial international and even intra‐national variation previously documented in both *H. pylori* prevalence and BQ consumption patterns [18].

## 5 Conclusion

In this cohort, male sex was the most robust, independent correlate of endoscopic pathology, significant across the primary model and all sensitivity analyses. Older age showed a modest, independent association in the primary model (*p* = 0.039) that did not persist in smaller sensitivity subgroups and should be interpreted cautiously. *H. pylori* infection, BQ use, their joint exposure, and a suggestive, non‐significant trend in ulcer/erosion prevalence with longer chewing‐session duration (*p* = 0.054) for BQ chewing (trend *p* = 0.054–0.076) each showed consistent but individually non‐significant, exploratory associations that require confirmation in adequately powered prospective studies.

## Author Contributions

**Mostak Uddin Ahmed**: conceptualisation, methodology, investigation, data curation, writing – original draft. **Sumona Tanu**: methodology, writing – review and editing. **Iftekhar Ahmad**: conceptualisation, methodology, validation, writing – review and editing. **Md. Jahangir Alam**: conceptualisation, methodology, validation, writing – review and editing. **Maisha Tanzim Mithila**: data curation, writing – review and editing. **Rukaia Tasin Athina**: data curation, writing – review and editing. **Ratan Chandra Bhowmik**: data curation, writing – review and editing. **A. N. M. Ishraqul Haque**: investigation, validation, writing – review and editing. **Md. Asaduzzaman**: conceptualisation, formal analysis, visualisation, supervision, writing – original draft, writing – review and editing. All authors read and approved the final manuscript and agree to be accountable for all aspects of the work.

## Funding

The authors have nothing to report.

## Ethics Statement

This study was conducted in accordance with the ethical principles of the Declaration of Helsinki and all applicable local regulations. Ethical approval was obtained from the Institutional Ethical Review Board of Sylhet MAG Osmani Medical College, Sylhet‐3100, Bangladesh (SOMC/2025/209).

## Consent

Written informed consent was obtained from all participants before inclusion in the study. Participants were informed about the objectives of the study, voluntary participation, confidentiality of their information, and their right to withdraw from the study at any time without affecting their medical care.

## Conflicts of Interest

The authors declare no conflicts of interest.

## Permission to Reproduce Material From Other Sources

The manuscript does not contain any previously published figures, tables, or other copyrighted materials requiring permission.

## Supporting information




**Supporting File 1**: puh270437‐sup‐0001‐SuppMat.docx.

## Data Availability

The data supporting the findings of this study are available from the corresponding author upon reasonable request (Email: asad_somc@yahoo.com).
