# Supplementary material for: Helicobacter pylori, Betel Quid Chewing, and Endoscopic Pathology in Dyspeptic Patients: A Cross‐Sectional Analytical Study
Source: Public Health Chall. 2026 Nov 6;5(4):e70437. doi: 10.1002/puh2.70437 (PMC13742375; doi:10.1002/puh2.70437)
Supplement: Supplementary file 1 [file PUH2-5-e70437-s001.docx]

**Supplementary data**

***Manuscript: Helicobacter pylori, Betel Quid Chewing, and Endoscopic Pathology in Dyspeptic Patients: A Cross-Sectional Analytical Study***

**Table S1: H. pylori test concordance:**

| **RUT result** | **Stool antigen: Negative** | **Stool antigen: Positive** | **Total** |
| --- | --- | --- | --- |
| Negative | 92 | 10 | 102 |
| Positive | 30 | 79 | 109 |
| Total | 122 | 89 | 211 |

**Table S2: Endoscopic biopsy rates across patient groups**

| **Group** | **n** | **Biopsy performed** | **% biopsied** |
| --- | --- | --- | --- |
| Group A (pathology) | 141 | 102 | 72.3% |
| Group B (normal), H. pylori+ | 36 | 0 | 0.0% |
| Group B (normal), H. pylori− | 34 | 2 | 5.9% |

*Biopsy/histology was reserved almost exclusively for patients with a grossly visible lesion (Group A); among Group B's 2 biopsied patients (both H. pylori-negative), histology showed chronic gastritis in both.*

.

**Table S3: Missingness by principal variable (n=211):**

| **Variable** | **n missing** | **% missing** |
| --- | --- | --- |
| Age | 13 | 6.2% |
| Sex | 4 | 1.9% |
| Residence | 22 | 10.4% |
| BMI | 92 | 43.6% |
| H. pylori status (RUT and/or stool antigen) | 0 | 0.0% |
| BQ-user status (yes/no) | 20 | 9.5% |
| BQ duration, years (among BQ users, n=145) | 46 | 31.7% |
| BQ-Quid-Years index (among BQ users) | 50 | 34.5% |
| Chewing-session duration, min (among BQ users) | 29 | 20.0% |
| Tobacco inclusion in quid (among BQ users) | 4 | 2.8% |
| Smoking status | 61 | 28.9% |

**Table S4: Comparison of participants with complete vs missing BMI data**

| **Characteristic** | **BMI complete (n=119)** | **BMI missing (n=92)** | **p-value** |
| --- | --- | --- | --- |
| Age, median | 40 | 36 | 0.072 |
| Sex, % male | 46.2% | 60.9% | 0.084 |
| Group A (pathology), % | 67.2% | 66.3% | 1.000 |
| H. pylori positive, % | 52.1% | 62.0% | 0.196 |
| BQ user, % | 81.5% | 66.7% | 0.031 |
| Rural residence, % | 83.8% | 77.8% | 0.403 |

*Participants with and without BMI data did not differ in pathology-group status, H. pylori status, age, sex, or residence, arguing against outcome-related selection bias. They did differ modestly in BQ-user status (81.5% vs 66.7%, p=0.031); the BMI-adjusted sensitivity analysis should therefore be interpreted with some caution, as it may under-represent heavier betel-quid users.*
